# Brain-specific immune hub activation in paediatric focal lesional epilepsy: a case–control study

**DOI:** 10.1093/braincomms/fcag152

**Published:** 2026-04-27

**Authors:** Antonio G Gennari, Alexia Rossi, Thomas Sartoretti, Moritz Schwyzer, Dorottya Cserpan, Valerie Treyer, Martin W Huellner, Ruth O’Gorman Tuura, Georgia Ramantani, Michael Messerli

**Affiliations:** Department of Nuclear Medicine, University Hospital Zurich, 8091 Zurich, Switzerland; University of Zurich, 8006 Zurich, Switzerland; Department of Neuropediatrics, University Children's Hospital of Zurich, 8008 Zurich, Switzerland; MR-Research Centre, University Children's Hospital of Zurich, 8008 Zurich, Switzerland; Department of Nuclear Medicine, University Hospital Zurich, 8091 Zurich, Switzerland; University of Zurich, 8006 Zurich, Switzerland; Department of Nuclear Medicine, University Hospital Zurich, 8091 Zurich, Switzerland; University of Zurich, 8006 Zurich, Switzerland; Department of Nuclear Medicine, University Hospital Zurich, 8091 Zurich, Switzerland; University of Zurich, 8006 Zurich, Switzerland; Department of Neuropediatrics, University Children's Hospital of Zurich, 8008 Zurich, Switzerland; Department of Nuclear Medicine, University Hospital Zurich, 8091 Zurich, Switzerland; University of Zurich, 8006 Zurich, Switzerland; Department of Nuclear Medicine, University Hospital Zurich, 8091 Zurich, Switzerland; University of Zurich, 8006 Zurich, Switzerland; University of Zurich, 8006 Zurich, Switzerland; MR-Research Centre, University Children's Hospital of Zurich, 8008 Zurich, Switzerland; Children’s Research Centre, University Children's Hospital of Zurich, 8008 Zurich, Switzerland; University of Zurich, 8006 Zurich, Switzerland; Department of Neuropediatrics, University Children's Hospital of Zurich, 8008 Zurich, Switzerland; Children’s Research Centre, University Children's Hospital of Zurich, 8008 Zurich, Switzerland; Department of Nuclear Medicine, University Hospital Zurich, 8091 Zurich, Switzerland; University of Zurich, 8006 Zurich, Switzerland

**Keywords:** paediatric epilepsy, inflammation, FDG-PET, brain-specific immune hub, metabolic activity

## Abstract

There are still some gaps in understanding the role of inflammation in focal lesional epilepsy. Surgical specimens often contain activated leukocytes, usually thought to originate from the systemic circulation. However, the cerebrospinal fluid, meninges, and skull form a distinct brain-specific immune hub that also reacts to local signals. This study assessed immune hub activation adjacent to epileptogenic lesions in children with focal lesional epilepsy using 2-[^18^F]-fluoro-2-deoxy-D-glucose (FDG)-PET. Children with focal lesional epilepsy who underwent brain FDG-PET for presurgical assessment were compared to age- and sex-matched controls who had total-body FDG-PET for other clinical indications. Spherical regions of interest (ROIs) were placed on the skull, with a cubic ROI on the pons used as a reference. In epilepsy patients, skull ROIs were positioned adjacent to the perilesional hypometabolic defect and contralaterally. In non-epilepsy patients, ROIs mirrored those of epilepsy patients. Metabolic activity was measured as maximum and mean standardized uptake values (SUV_max_ and SUV_mean_). Corrected SUV (cSUV), normalized to the pons, and asymmetry index (AI), comparing lesional and contralateral ROIs, were calculated. Twenty-nine epilepsy and 29 non-epilepsy patients (16 boys, 55%) were included. The median age at the time of the scan was 9.0 years (interquartile range, IQR: 3.0–14.0). Across all patients, the median cSUV_max_ and cSUV_mean_ were 0.36 (IQR: 0.27–0.48) and 0.23 (IQR: 0.16–0.32), respectively. In epilepsy patients, cSUV_max_ and cSUV_mean_ were 0.33 (IQR: 0.26–0.40) and 0.23 (IQR: 0.18–0.31), and in non-epilepsy patients, 0.44 (IQR: 0.29–0.55) and 0.23 (IQR: 0.13–0.33). Neither cSUV_max_ nor cSUV_mean_ differed by side (*W* = 800, *P*: 0.84; *W* = 682, *P*: 0.25), confirming comparability. However, in epilepsy patients, both cSUV_max_ and cSUV_mean_ were higher on the lesional side than on the contralateral side (*W* = 43 and *W* = 49, *P* < 0.001 for both). In non-epilepsy patients, mirrored ROIs showed no significant difference (*W* = 142, *P*: 0.17; *W* = 154, *P*: 0.27). AI values for SUV_max_ and SUV_mean_ were higher in epilepsy than in non-epilepsy patients (*t* = −4.36 and *t* = −3.58, both *P* < 0.001), a difference that remained significant after covariate adjustment, demonstrating metabolic asymmetry relative to the epileptogenic lesion. In children with focal lesional epilepsy, we observed increased metabolic activity in the brain-specific immune hub adjacent to the epileptogenic lesion. This local immune activation likely plays a role in the disease mechanism, which may clarify why immune-modulating treatments can be effective and point the way towards new therapeutic approaches.

## Teaser

Gennari *et al*. report a 2-[^18^F]-fluoro-2-deoxy-D-glucose-positron emission tomography-based case–control study of children with focal lesional epilepsy and age- and sex-matched controls. They observed increased metabolic activity in skull regions adjacent to epileptogenic lesions, indicating activation of a brain-specific immune hub that may contribute to epileptogenesis and represent a novel therapeutic target.

## Graphical Abstract

**Graphical Abstract:**
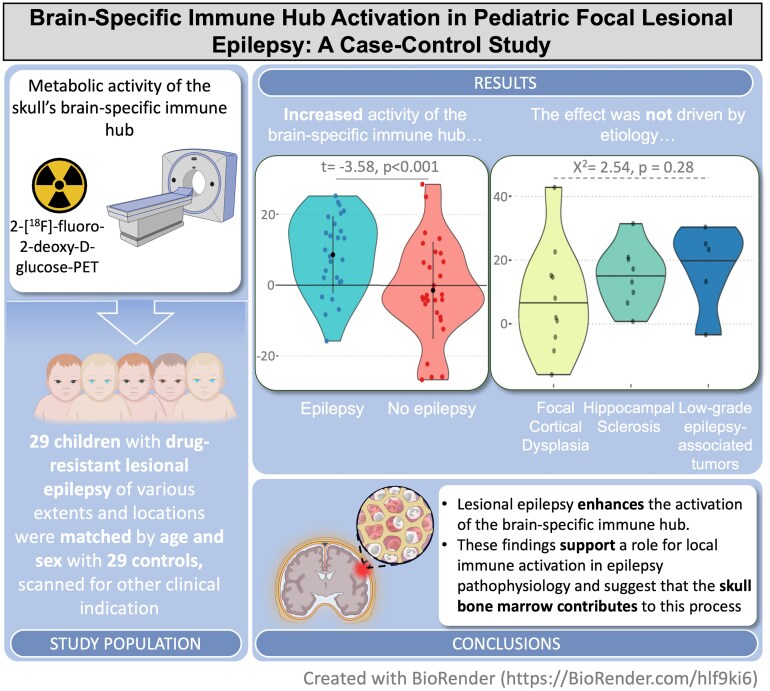

## Introduction

Emerging evidence highlights the role of inflammation in initiating and sustaining seizures,^1,2^ as indicated by the elevated uptake of neuroinflammation-specific radiotracers ipsilateral to the epileptogenic zone (EZ) in nuclear medicine studies.^3,4^ Inflammatory mediators and activated leukocytes have also been found in resected tissue from patients with focal lesional epilepsy undergoing epilepsy surgery, though their origin and role remain unclear.^1,5^ Paediatric studies have linked cerebrospinal fluid (CSF) cytokine and chemokine levels to epilepsy severity^6^; similarly, systemic markers such as plasma cytokine profiles have shown a pro-inflammatory pattern in temporal lobe epilepsy that normalized after surgery.^7^ Recent findings indicate that the CSF, meninges, and skull bone marrow form a dedicated immune hub that dynamically reacts to local brain signals.^8-10^ Moreover, CSF-derived pathogens and brain signals interact with immune niches in the skull bone marrow via transdural arachnoid granulations, lymphatic pathways, and ossified skull channels.^11-13^ MRI studies further confirmed connections between CSF and the skull’s bone marrow,^14^ reinforcing the role of this immune hub in brain pathology. This network not only provides an additional route for CSF drainage^9,15^ but also facilitates immune cell trafficking under both homeostatic and inflammatory conditions, thereby bypassing the blood–brain barrier.^8,9,11,12,16^

2-[^18^F]-fluoro-2-deoxy-D-glucose (FDG)-PET is an established component of presurgical evaluation^17^ for localizing the EZ in focal lesional epilepsy,^18-23^ particularly when EEG or MRI findings are inconclusive or discordant.^18-21,22^ Beyond its application in epilepsy, FDG-PET has been widely used to detect infection and inflammation in various disorders.^24^ Interestingly, neuroinflammation-specific PET tracers have identified skull and meningeal inflammation in adults with conditions such as migraine, depression, Alzheimer’s disease, and multiple sclerosis.^25-27^ However, the connection between focal lesional epilepsy and immune hub activation in the skull bone marrow remains unexplored, particularly in children, whose developing immune system may amplify such responses. Understanding this relationship could have significant implications for treatment, potentially guiding novel therapeutic strategies.

This proof-of-concept study sought to compare the metabolic activity of skull bone marrow in children with focal lesional epilepsy to that of age- and sex-matched controls, hypothesizing that local inflammatory responses associated with epilepsy would activate the brain-specific immune hub formed by the CSF, meninges, and skull bone marrow.

## Materials and methods

### Study population

This exploratory study compared two cohorts:

- *Epilepsy patients:* Children with focal lesional epilepsy who underwent FDG-PET for presurgical evaluation.
- *Non-epilepsy patients:* Children who underwent total-body FDG-PET for other clinical indications.

### Epilepsy patients

Τhis retrospective, single-centre study included children evaluated for epilepsy surgery at the University Children’s Hospital Zurich who underwent brain FDG-PET at the University Hospital of Zurich between 1 January 2005 and 31 May 2021. Inclusion criteria were: (i) age ≤18 years at the time of FDG-PET, (ii) diagnosis of focal lesional epilepsy based on electroclinical correlations and MRI findings, (iii) epileptogenic cortical lesion on MRI or histopathology, and (iv) FDG-PET findings consistent with electroclinical and MRI results. Exclusion criteria were: (i) infratentorial lesions, (ii) extensive complex multilobar or hemispheric brain lesions (e.g. cortical malformations with corpus callosum atrophy, intracranial cysts or perinatal stroke), (iii) prior resective epilepsy surgery, (iv) concomitant genetic defects associated with epilepsy, indicating diffuse bilateral epileptogenicity beyond the epileptogenic lesion as defined by presurgical evaluation, and (v) poor image quality. Epileptogenic lesions were classified by hemisphere (right, left, or bilateral) and lobe (frontal, temporal, parieto-occipital, or multilobar). Since lesion dimensions may impact functional imaging results,^28,29^ lesions were categorized as small or medium-to-large. Small lesions included those spanning two or fewer gyri, including the sulcus between them, and less than half a gyrus in length; all others were categorized as medium-to-large.^30^ Lesion location was defined as deep when affecting the mesial or basal part of the frontal, parietal, occipital, or temporal lobes, or the insula. All other lesions were classified as superficial. In MRI-negative cases, the location was inferred from surgical reports and post-surgical imaging of the resection cavity.

### Non-epilepsy patients

Epilepsy patients were 1:1 matched with non-epilepsy patients of the same age and sex who underwent total-body FDG-PET for other clinical indications, such as malignancy, fever of unknown origin, or infection, during the same period. FDG-PET findings and medical history were reviewed to assess the disease state at the time of imaging and long-term outcome. Exclusion criteria included: (i) history of epileptic seizures or anti-seizure medication (ASM) use, (ii) primary brain malignancies, (iii) brain or skull metastatic involvement, (iv) systemic diseases with known CNS involvement, and (v) prior brain/head surgery, irradiation, or major trauma. Patients with haematological tumours receiving chemotherapy were not excluded, as studies suggest that skull immune cells originate independently of systemic immune circulation and are resistant to such interventions.^31^ Although controls were not healthy children, this approach has been methodologically validated in previous studies.^32^

The study was approved by the local ethics committee (2020-03067) and was conducted in compliance with International Council for Harmonisation-Good Clinical Practice rules and the Declaration of Helsinki. Written informed consent was waived for patients whose scan was acquired before January 2016. After January 2016, written informed consent to reuse clinical data for research purposes was obtained from all patients and their parents/legal guardians.

### Matching technique

Due to the limited sample size, epilepsy and non-epilepsy patients were manually matched 1:1 using exact matching without replacement, prioritizing sex and age to ensure cohort independence.^33,34^ Since the FDG dose is weight-based, weight proximity was also considered.^35^

### Image acquisition

A subset of included patients has been reported in our previous studies.^17,36^  Supplementary Table 1 details the FDG-PET scanners used (all from GE HealthCare, Waukesha, WI). In all cases, computed tomography (CT) or MRI was used for PET attenuation correction and anatomical co-localization.

### PET acquisition

All patients fasted for at least 4 h before FDG injection to optimize tracer uptake. In epilepsy patients, an in-house, weight-based three-tier protocol determined the injected FDG dose^17^: (i) children <15 kg received a fixed dose of 43.1 MBq, (ii) children 15–60 kg received a weight-adjusted dose, and (iii) children >60 kg received a fixed dose of 100 MB.^17^ The uptake time was standardized at 50–60 min. In non-epilepsy patients, until 2016, injected doses followed a body weight-based standardized protocol. From 2017 onward, dosing adhered to European Association of Nuclear Medicine guidelines, using a body mass index-based protocol.^36^ The uptake time was standardized at 60 min. Protocol deviations were allowed based on clinical requirements. Sedation was administered as clinically indicated.

In epilepsy patients, CT scans were acquired after PET in PET/CT, while MRI scans were acquired simultaneously in PET/MRI, with coverage limited to the skull. In non-epilepsy patients, CT and MRI images were always acquired before PET, with coverage extending from the skull to the mid-thighs. Supplementary Table 2 lists the PET reconstruction algorithms.

### Image analysis

All images were analysed using Advantage Workstation (Version 4.7, GE HealthCare) by a fully trained radiologist with 10 years of neuroradiology experience (A.G.G.).

In epilepsy patients, FDG-PET images were inspected to locate the metabolic defect corresponding to the epileptogenic lesion. Using anatomical images, two semi-automated regions of interest (ROIs) were drawn to ensure precise placement:

- Skull ROI: A spherical ∼13 mm^3^ ROI was placed on the skull adjacent to the hypometabolic brain area and the contralateral homotopic skull^25,26^ (Supplementary Fig. 1). The ROI size was standardized to ensure uniform dimensions between flat and non-flat bones and was always centred on the skull diploe adjacent to the inner compact bone layer. For temporal metabolic defects, ROIs were placed on the greater wing of the sphenoid bone.
- Pons ROI: A cubic 8 mm^3^ ROI was placed in the anterior pons^37^ (Supplementary Fig. 1).

In non-epilepsy patients, ROI sizes and locations were identical to those of epilepsy patients.^34^

Maximum and mean standardized uptake values (SUV_max_ and SUV_mean_) were derived from all ROIs. Given the use of multiple scanners, reconstruction algorithms, and injection protocols over time, SUV values were normalized using the corrected SUV (cSUV) and asymmetry index (AI)^3^ as follows:


cSUV=(SkullSUV)/(PonsSUV)

$$\mathrm{AI}=\left(\frac{\mathrm{Skull}\;\mathrm{SU}V_{\mathrm{ipsilateral}}-\mathrm{Skull}\;\mathrm{SU}V_{\mathrm{contralateral}}}{\mathrm{Skull}\;\mathrm{SU}V_{\mathrm{ipsilateral}}+\mathrm{Skull}\;\mathrm{SU}V_{\mathrm{contralateral}}}\right)\times 100$$

### Intrareader variability

To check the intrareader variability, 1 month after the initial analysis, the same reviewer reanalysed the images of 24 randomly selected children (14 epilepsy and 10 non-epilepsy).^38^

### Statistical analysis

Statistical analysis was performed using R software (version 4.4.2, https://www.r-project.org). Normality was assessed using the Shapiro–Wilk test. Continuous variables were reported as mean ± standard deviation (SD) or median (interquartile range, IQR), while categorical variables were reported as frequencies and percentages. Comparisons were performed using the Chi-square or Fisher’s exact test for categorical variables, whereas group-wise comparison for continuous variables was performed with two-tailed *parametric or non-parametric tests* (e.g. *t-*test or Welch *t-*test, Kruskal–Wallis’s, Wilcoxon–Mann–Whitney). Correlations were assessed using Spearman’s coefficient. Intrareader variability was evaluated using the intraclass correlation coefficient and its confidence interval (CI).

Before testing the main hypothesis, potential confounders were explored, including within-group and between-group differences in cSUV_max_ and cSUV_mean_, and the effects of sedation,^39^ sex,^40,41^ and pubertal status on cSUV values. Puberty was estimated using age cut-offs: ≥9 years for females and ≥10 years for males.^42^ Cases with missing sedation data were classified as sedated if their age at the time of the scan was <6 years.

The Student’s *t-*test was first used to compare AI values (derived from SUV_max_ and SUV_mean_) between epilepsy and non-epilepsy patients. Then, multivariate linear regression adjusted for matching covariates (age and sex) and pubertal status was performed.^34^ Sensitivity analysis using the *E*-value^43^ was conducted to assess robustness against unmeasured confounding. Differences across histologies and clinical diagnoses (≥4 cases) were also explored.

Statistical significance was set at *P* ≤ 0.05, with Holm correction applied for multiple comparisons.

## Results

### Study cohort

**Figure 1:**
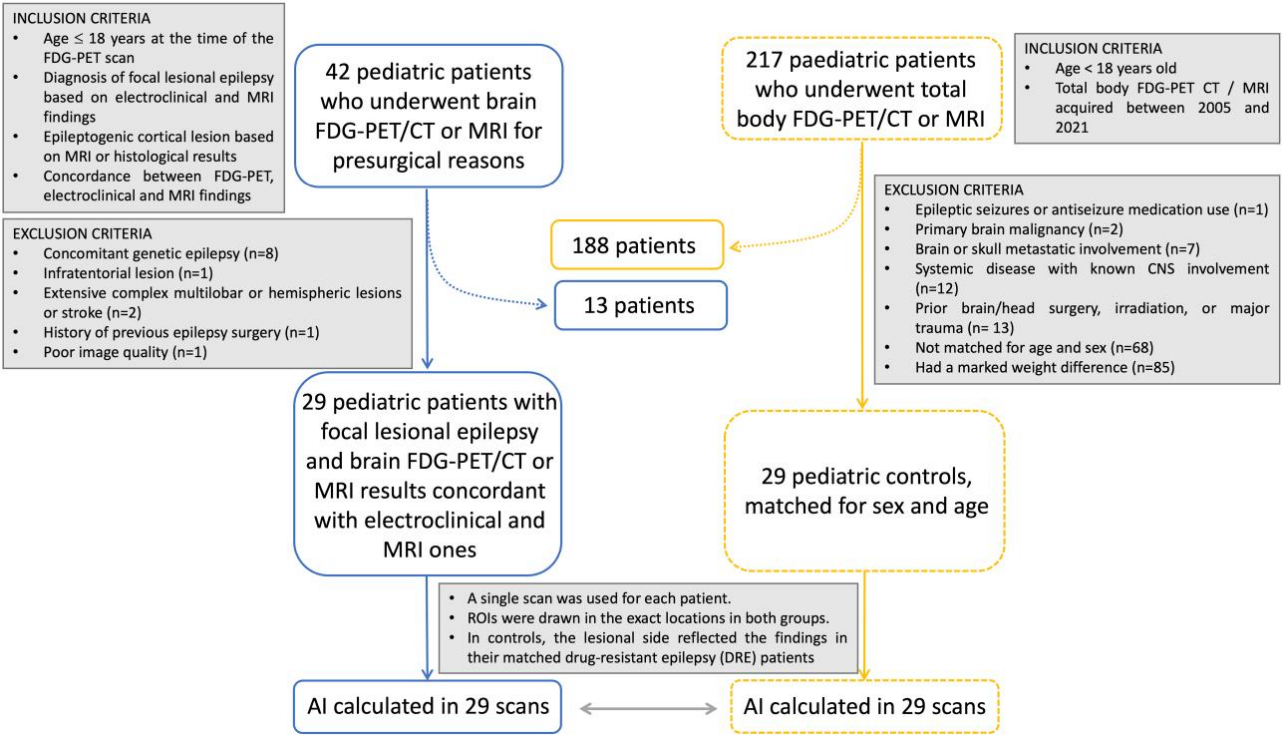
**Patient selection workflow.** CNS: central nervous system; CT: computed tomography; FDG: 2-[^18^F]-fluoro-2-deoxy-D-glucose; MRI: magnetic resonance imaging; PET: positron emission tomography; ROI: region of interest.

Of the 42 epilepsy patients initially considered, 13 were excluded (Fig. 1). The final cohort comprised 29 epilepsy and 29 non-epilepsy patients (16 boys, 55%), with a median age of 9.0 years (IQR: 3.0–14.0) at the time of the scan (Tables 1 and 2). Sedation was used in 20 cases (34%), including 12 epilepsy patients. Sedation status was unavailable in six cases (all non-epilepsy patients). Two of these children, aged 0 and 2 years, were thus classified as sedated in further analyses. Among the 20 sedated children with known sedation protocols, the most common regimens were propofol with ketamine (40%) and propofol alone (30%). The remaining cases received propofol with ketamine and nalbuphine (three cases), propofol with nalbuphine (two cases), or propofol with an analgesic (one case).

**Table 1:** Patients’ characteristics

	Epilepsy patients (*n* = 29)	Non-epilepsy patients (*n* = 29)	*P*-value
Male, *n* (%)	16 (55%)	16 (55%)	1^a^
Age at epilepsy onset, y^b^	4.8 (0.9–10.5)		
Epilepsy duration, y^b^	2.1 (1.2–3.8)		
Age at the time of the scan, y^b^	9.0 (3.0–14.0)	9.0 (3.0–14.0)	1^c^
Weight at the time of the scan, kg^b^	31 (17–60)	32 (15–56)	0.5^c^
PET technique, *n* (%)			0.4^d^
FDG-PET/CT	18 (62%)	21 (72%)	
FDG-PET/MRI	11 (38%)	8 (28%)	
Injected dose, MBq^b^	95 (64–100)	124 (89−265)	<0.001^c^
Administered activity per Kg body weight, MBq/Kg^b^	2.2 (1.7–3.7)	4.6 (2.6–7.5)	<0.001^c^

^a^Chi-squared test.

^b^Data presented as median (interquartile range).

^c^Wilcoxon rank sum test.

^d^Fisher’s exact test. CT: computed tomography; FDG: 2-[^18^F]-fluoro-2-deoxy-D-glucose; Kg: kilogram; IQR: interquartile range; MBq: mega becquerel; MR: magnetic resonance imaging; PET: positron emission tomography; SD: standard deviation; y: years.

**Table 2:** Detailed underlying aetiologies in epilepsy and non-epilepsy patients

Diagnosis, *n* (%)			
Epilepsy patients		Non-epilepsy patients	
Focal cortical dysplasia	10 (34%)	Hodgkin lymphoma^a^	12 (39%)
Hippocampal sclerosis	8 (28%)	Granulomatous disorder^b^	6 (20%)
Low-grade epilepsy-associated tumours	5 (17%)	Unspecified infectious disease^c^	3 (10%)
Sturge Weber	2 (7%)	Follicular lymphoma^d^	3 (10%)
Rasmussen’s encephalitis	2 (7%)	Malignant neoplasms of bone and articular cartilage^e^	2 (6%)
Tuberous sclerosis	2 (7%)	Malignant neoplasm of connective and soft tissue^f^	1 (3%)
		Melanoma and other malignant neoplasm of the skin^f^	1 (3%)
		Malignant neoplasm of liver and intrahepatic bile duct^f^	1 (3%)

^a^Eight of 12 (67%) Hodgkin lymphoma cases were in partial or complete remission at the time of the scan, while the remaining (33%) were newly diagnosed.

^b^Three of six (50%) granulomatous disorder cases were scanned due to fever, while the other three (50%) underwent routine follow-up, with one already in remission.

^c^Two of three unspecified infectious disease cases were scanned due to fever, while the third underwent routine follow-up.

^d^Two of three follicular lymphoma cases were in complete remission at the time of the scan, while the remaining one was scanned due to fever.

^e^The bone tumour cases included one chondroblastoma and one Ewing sarcoma: the latter showed no signs of metastasis at 5-year follow-up.

^f^These cases had the following conditions: an inflammatory myofibroblast tumour, a melanoma with no metastasis at 18-year follow-up and a hepatoblastoma.

### Epilepsy patients

All epilepsy patients had an epileptogenic brain lesion (Table 2), which was left-sided in 18 cases (62%). Lesion localization was temporal in 16 cases (55%), frontal in 5 cases (17%), posterior in 2 cases (7%), and multilobar in 6 cases (21%). Nineteen lesions (66%), including two MRI-negative cases, were classified as small, and 15 (52%) were located deeply. The median age at epilepsy onset was 4.8 years (IQR: 0.9–10.5), while the median epilepsy duration was 2.1 years (IQR: 1.2–3.8). At the time of the scan, 13 patients (45%) were on a single ASM, 9 (31%) were on two ASMs, and 7 (24%) were on three or more ASMs. Twenty-four of 29 epilepsy patients (83%) eventually underwent resective surgery. The histology findings in operated patients were focal cortical dysplasia in 10 cases (42%), hippocampal sclerosis in 6 cases (25%), low-grade epilepsy-associated tumour in 4 cases (17%), Rasmussen’s encephalitis and Sturge-Weber syndrome in 2 cases (8%) each (Table 2). Among the five non-operated patients, two each had tuberous sclerosis and hippocampal sclerosis, and one had a low-grade epilepsy-associated tumour.

### Non-epilepsy patients

Among non-epilepsy patients, the most common clinical diagnoses at the time of the scan were Hodgkin lymphoma in 12 cases (41%), granulomatous disorder in 6 cases (21%), unspecified infectious disease and follicular lymphoma in 3 cases (10%) each (Table 2). At the time of the PET scan, 10 patients (45%) were in complete remission (8 haematological malignancies) or partial remission (3 Hodgkin lymphoma), all of whom later achieved complete remission. Of the remaining cases, four (14%, all Hodgkin lymphoma) were at their first diagnosis, while six (21%) underwent FDG-PET for fever of unknown origin. The remaining six cases underwent FDG-PET for following up granulomatous disorders and solid tumours in three cases (10%) each.

### Administered activity and cSUV measurements

Due to the different injection protocols, epilepsy patients received a significantly lower mean FDG activity per body weight than non-epilepsy patients (*W* = 651, *P*  *<* 0.001, Wilcoxon–Mann–Whitney, Table 1). The median cSUV_max_ and cSUV_mean_ across all patients were 0.36 (IQR: 0.27–0.48) and 0.23 (IQR: 0.16–0.32, Supplementary Fig. 1). Group-specific values were cSUV_max_: 0.33 (IQR: 0.26–0.40) and cSUV_mean_: 0.23 (IQR: 0.18–0.31) for epilepsy patients and cSUV_max_: 0.44 (IQR: 0.29–0.55) and cSUV_mean_: 0.23 (IQR: 0.13–0.33) for non-epilepsy patients. cSUV_max_ correlated with FDG activity per body weight (*r_s_*: 0.26, *P*: 0.004), whereas cSUV_mean_ did not (*r_s_*: −0.08; *P*: 0.37, Supplementary Fig. 2A and B). No significant differences were detected in the overall cSUV_max_ or cSUV_mean_ by measurement side (*W* = 800, *P*: 0.84 and *W* = 682, *P*: 0.25, respectively, Wilcoxon–Mann–Whitney). Similarly, no differences were observed in homotopic skull ROIs between epilepsy and non-epilepsy patients (Table 3).

**Table 3:** Corrected standardized uptake value (cSUV) by cohort and lateralization

	Epilepsy patients (*n* = 29)	Non-epilepsy patients (*n* = 29)	*W*-value	*P*-value
cSUV_max_^a^				
Left	0.33 (0.26–0.38)	0.45 (0.29–0.51)	579	0.06
Right	0.31 (0.26–0.40)	0.43 (0.29–0.61)	554	0.11
cSUV_mean_^a^				
Left	0.25 (0.14–0.34)	0.24 (0.19–0.32)	387	1
Right	0.23 (0.13–0.31)	0.21 (0.17–0.28)	423	1

^a^Data are presented as median (IQR). IQR: interquartile range; cSUV: corrected standardized uptake value.

**Figure 2:**
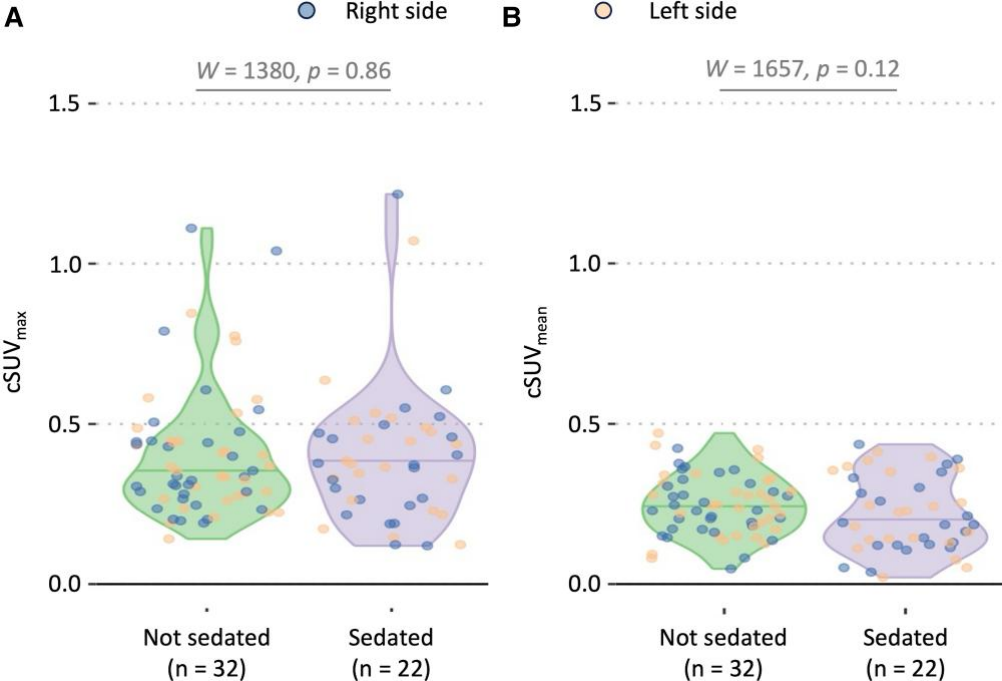
**Sedation did not impact cSUV values.** Violin plots show the overall distribution of (**A**) cSUV_max_ and (**B**) cSUV_mean_ values based on sedation status (Wilcoxon–Mann–Whitney). In total, 22 cases were scanned under sedation, 32 without sedation, and the sedation status was unknown for the remaining four (not included in the analysis). Of the 22 sedated patients, sedation protocol was known in 20, while the remaining two had an age at scan <6 years of age. Each violin plot includes a horizontal line indicating the median cSUV value. Left (orange) and right (blue) cSUV values are differently coloured to enhance the graph’s readability. cSUV: corrected standardized uptake value.

cSUV_max_ and cSUV_mean_ were not affected by pubertal status (*W* = 1762, *P*: 0.65 for cSUV_max_ and *W* = 1440, *P*: 0.19 for cSUV_mean_, Wilcoxon–Mann–Whitney, Supplementary Fig. 3A and B) or sedation status (*W* = 1380, *P*: 0.86 for cSUV_max_ and *W* = 1657, *P*: 0.12 for cSUV_mean_, Wilcoxon–Mann–Whitney, Fig. 2).

In epilepsy patients, both cSUV_max_ and cSUV_mean_ were significantly higher on the lesional side than the contralateral side (*W* = 43 and *W* = 49, respectively, *P* < 0.001 for both, Wilcoxon–Mann–Whitney). In contrast, non-epilepsy patients (mirrored ROIs), cSUV_max_ and cSUV_mean_ showed no side differences (*W* = 142, *P*: 0.17 and *W* = 154, *P*: 0.27, respectively).

### Intrareader variability

The intraclass correlation coefficient was 0.77 (CI: 0.59–0.87) for cSUV_max_ and 0.81 (CI: 0.66–0.89) for cSUV_mean,_ indicating good reliability of our measures.

### AI analysis

**Figure 3:**
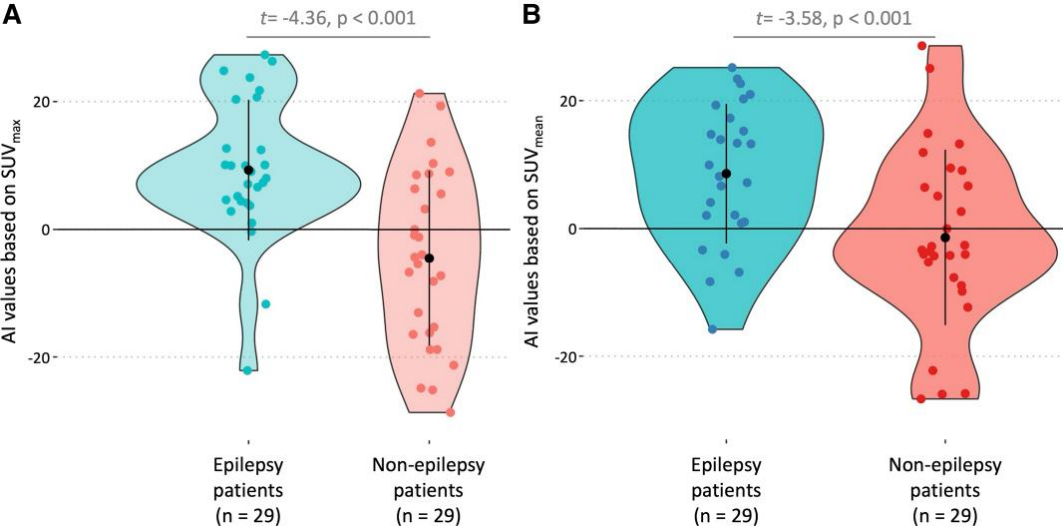
**Increased skull bone marrow metabolic activity adjacent to the epileptogenic lesions.** Violin and dot plots illustrating AI differences, calculated using (**A**) SUV_max_ and (**B**) SUV_mean_, between epilepsy patients and non-epilepsy patients (Welch *t-*test). AI values were significantly higher in epilepsy patients, suggesting increased metabolic asymmetry in the skull bone marrow adjacent to the epileptogenic lesions. Black dots and lines indicate mean values and standard deviations. Blue and red dots represent epilepsy patients and non-epilepsy patients. Light colours correspond to AI values derived from SUV_max_, while solid colours indicate values computed using SUV_mean_. AI: asymmetry index; FDG: 2-[^18^F]-fluoro-2-deoxy-D-glucose; SUV: standardized uptake value.

**Figure 4:**
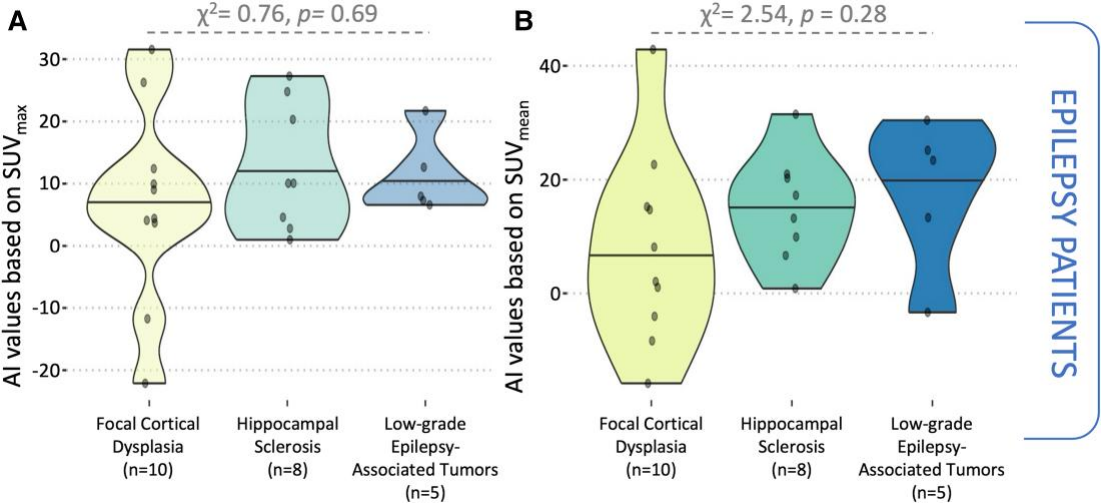
**AI values did not differ by histological diagnosis.** Violin plots illustrating the distribution of AI values by histological diagnoses in epilepsy patients. The number of cases and corresponding histological diagnoses are indicated below each violin. Based on the Kruskal–Wallis test, no significant differences were observed in AI values based on (**A**) SUV_max_ (χ^2^ = 0.76, *P*: 0.69) or (**B**) SUV_mean_ (and χ^2^ = 2.54, *P*: 0.28).

AI values were significantly higher in epilepsy patients than in non-epilepsy patients for both SUV_max_ (10 ± 11.6 versus −4.5 ± 13.7, *t* = −4.36, *P* < 0.001, Welch *t-*test) and SUV_mean_ (11.3 ± 13.3 versus −1.4 ± 13.7, *t* = −3.58, *P* < 0.001, Welch *t-*test, Fig. 3). These differences remained significant after adjusting for age, sex and pubertal status [*F*(4, 53) = 4.86, *P*: 0.002 for SUV_max_ and *F*(4, 53) = 3.56, *P*: 0.01 for SUV_mean_, Table 4]. To nullify the observed effect, an unmeasured confounder would need to increase the risk ratio of both epilepsy and lateralized hypermetabolism by a factor of four (*E*-values: SUV_max_ = 4.4 and SUV_mean_ = 3.8). Furthermore, no significant histology-based differences in AI values were detected among epilepsy patients (χ^2^ = 0.76, *P*: 0.69 and χ^2^ = 2.54, *P*: 0.28, Kruskal–Wallis, Fig. 4A and B).

**Table 4:** Multivariable linear regression models for epilepsy patients, adjusted for age, sex and pubertal status

	Estimate	Confidence intervals	*t*-value	*P*-value
				
AI based on SUV_max_	14.5	7.7 to 21.3	4.3	**<0**.**001**
Sex (male)	2.4	−4.5 to 9.3	0.7	0.49
Age, y	−0.2	−1.7 to 1.3	−0.2	0.84
Pubertal status (puberty)	−1.1	−17.1 to 15.2	−0.1	0.89
				
AI based on SUV_mean_	12.7	5.5 to 19.9	3.5	**<0**.**001**
Sex (male)	2.2	−5.1 to 9.5	0.6	0.54
Age, y	−0.8	−2.4 to 0.8	−1.0	0.31
Pubertal status (puberty)	5.5	−11.8 to 22.8	0.6	0.52

The bold values in the table indicate statistically significant variables. AI: asymmetry index; SUV: standardized uptake value; y: years.

In epilepsy patients, AI values derived from SUV_max_ and SUV_mean_ did not correlate with age at epilepsy onset (*r_s_*: −0.31, *P*: 0.10, and *r_s_*: −0.18, *P*: 0.34) or epilepsy duration (*r_s_*: 0.06, *P*: 0.77, and *r_s_*: −0.08, *P*: 0.68), nor were they affected by lesion size (*t* = 0.64, *P* = 0.53, and *t* = −0.82, *P* = 0.41, Welch *t-*test) or location (*t* = −0.12, *P* = 0.90, and *t* = −0.93, *P* = 0.36, Welch *t-*test, Supplementary Fig. 4A–D).

## Discussion

This highly innovative proof-of-concept study, performed in a paediatric population, is the first to demonstrate a link between FDG-PET findings and activation of a brain-specific immune hub. Specifically, AI values were significantly higher in epilepsy than in non-epilepsy patients, indicating increased metabolic activity in the brain-specific immune hub adjacent to the EZ. The consistency of cSUV_max_ and cSUV_mean_ values across measurements confirmed the reliability of our metabolic assessments. The lack of AI differences across epilepsy substrates suggests that skull bone marrow activation is EZ-specific rather than lesion-specific.

Traditionally, the brain has been considered immune-privileged due to its distinct immune responses and the absence of a classical lymphatic system.^44^ However, recent evidence highlights the role of the skull bone marrow, meninges, and CSF as a functional neuroimmune interface capable of mounting both innate and adaptive immune responses. Animal studies show that skull bone marrow niches respond to neuroinflammation^45^ and that haematopoietic cells from different bone marrow locations, including the skull and tibia present similar properties, suggesting site-independent immune reactivity.^45^

Our observation aligns with studies reporting diploic hypermetabolism in multiple sclerosis, depression, Alzheimer’s disease, and migraine patients.^25-27^ The known pathophysiological overlap between epilepsy and neurodegenerative diseases, such as amyloidogenesis and tau phosphorylation triggered by epileptiform activity and seizures in dementia,^37^ supports the translational relevance of these findings. The smaller effect size in our study, compared with that observed in neurodegenerative conditions, may reflect differences in radiotracers or a lower inflammatory burden in focal lesional epilepsy. While systemic immune activation in epilepsy is well documented, our findings suggest that some of the immune cells observed in epilepsy surgery specimens may originate from the skull bone marrow and meningeal immune hubs. In stroke models,^26^ monocytes and neutrophils from the skull infiltrate the dura through trans-osseous connections,^31^ probably in response to increased protein exchange between the calvaria and the brain.

Neuroinflammation plays a key role in epilepsy, spanning the acute, subacute, and chronic phases.^46^ Epilepsy is considered a model of sterile inflammation, with elevated proinflammatory cytokines such as interleukin-1β (IL-1β) and tumour necrosis factor-α (TNF-α), as well as activated microglia, astrocytes, and myeloid cells in lesional tissue.^1^ Based on previous animal studies,^9^ we hypothesize that solutes, inflammatory cytokines and waste from inflamed neurons and reactive astrocytes^47^ are cleared via the para-venous glymphatic route into the CSF.^11^ These signals may then reach the neuroimmune interface at the peri-sinus region,^14^ triggering local leukocyte recruitment in the skull bone marrow.^26,48^ This may explain the delayed onset of monocyte infiltration relative to blood–brain barrier breakdown observed in previous studies.^47^ Although we did not establish a direct link between immune activation and seizure generation, our findings strengthen the association between epileptogenic lesions and adjacent immune activity.

This finding has several potential clinical implications for the diagnosis and treatment of focal structural epilepsy (i) improving presurgical evaluation by refining lateralization and localization of the EZ, and (ii) exploring immune-modulating interventions. Skull metabolism may serve as a complementary biomarker. However, validation with advanced voxel-wise mapping is needed to quantify and localize metabolic asymmetry between lesional and contralateral regions. This kind of analysis may also evaluate whether activation patterns in the skull immune hub present metabolic gradient alterations as observed in FDG-PET perilesional metabolic changes.^49^

Anti-inflammatory treatments, such as systemic steroids and local cortical cooling, are already in clinical use in epilepsy. Targeting immune responses within the skull bone marrow could provide a novel strategy for seizure control. In a recent study of patients with highly refractory epilepsy, 40% achieved seizure reduction after empiric immunotherapy, with 30% achieving >50% seizure reduction.^50^ Interestingly, MRI-positive patients had higher response rates than MRI-negative patients,^50^ supporting a link between neuroinflammation and epileptogenesis, as well as a potential role for immune-modulating therapy in focal structural epilepsy.

We found no correlation between AI values and epilepsy duration or age at onset, suggesting that immune hub activation is independent of disease chronicity (Supplementary Fig. 4). This is consistent with findings in patients with mesial temporal sclerosis, where neuroinflammation-specific PET uptake did not correlate with seizure frequency or epilepsy duration.^3^ Similarly, AI values did not differ across histological subtypes, possibly due to limited sample size. Of note, given that epileptogenic lesions typically exhibit hypometabolism rather than hypermetabolism on FDG-PET, a spill-in effect from the brain to the skull is also unlikely.

Moreover, our results showed no significant differences in cSUV values between sedated and non-sedated patients. While propofol is known to reduce microglial activation, suppress proinflammatory cytokine secretion^51^ and lower global brain metabolism,^52^ it does not appear to influence skull bone marrow activity in this context. The limited sedation duration likely minimized its impact on the skull bone marrow activity.

Sex-based differences have been reported in both innate^53^ and post-stroke^54^ brain immunity, with higher neuroinflammatory responses noted in female Alzheimer’s disease patients.^26^ However, our study showed no pubertal effects on immune activation (Supplementary Fig. 3). Larger studies with longitudinal sampling are needed to explore age- and sex-related hormonal influences on immune hub activity, as well as the clinical impact of different grades of immune hub activation.

### Limitations

While the clear inclusion criteria, careful matching of patients with controls, ROI standardization, and thorough analyses of potential confounders are the main strengths of this proof-of-concept study, some limitations must still be acknowledged. First, our non-epilepsy group primarily consisted of children with clinical indications for FDG-PET, often neoplastic disease. While true healthy controls were not available due to ethical constraints, this approach is supported by prior studies using oncological patients, such as those with lymphoma, as extracranial references.^32^ A sensitivity analysis indicated that an unmeasured confounder would need to be strongly associated with both epilepsy and skull bone marrow activity to negate the observed findings. Second, our study lacks histological confirmation of immune activation within the skull bone marrow. While inflammation-specific tracers or immunohistochemical analysis of human skull tissue would provide definitive evidence, both approaches were unfeasible in this retrospective paediatric study. Third, the retrospective design led to the use of different scanners and imaging protocols (Supplementary Tables 1 and 2). However, this was partially mitigated by the comparison of intrasubject SUV values. Finally, the impact of lesion size on skull immune activity was only subjectively quantified. Future animal studies could provide quantitative, histological, and mechanistic insight into this relationship.

## Conclusions

This proof-of-concept study demonstrates increased metabolic activity in the brain-specific immune hub near the epileptogenic lesion in children with focal lesional epilepsy. These findings support a local immune activation in epilepsy pathophysiology and suggest that skull bone marrow may contribute to this process. However, further studies are needed to corroborate our preliminary findings with molecular and histological data and to explore whether modulation of this immune hub could lead to new anti-inflammatory and anti-seizure therapies.

## Supplementary Material

fcag152_Supplementary_Data

## Data Availability

The datasets generated during and/or analysed during the current study are not publicly available because they are based on diagnostic images. Nonetheless, they are available from the corresponding author upon reasonable request.
