# Supplementary material for: Brain-specific immune hub activation in paediatric focal lesional epilepsy: a case–control study
Source: Brain Commun. 2026 Apr 27;8(3):fcag152. doi: 10.1093/braincomms/fcag152 (PMC13201093; doi:10.1093/braincomms/fcag152)
Supplement: fcag152_Supplementary_Data [file fcag152_supplementary_data.docx]

***Supplementary Materials***

**Brain-specific immune hub activation in pediatric focal lesional epilepsy: a case-control study**

**Summary**

[Supplementary Table 1 – Different scanners used in the study 2](#_Toc194669462)

[Supplementary Table 2 – Different reconstruction algorithms used in the study 2](#_Toc194669463)

[Supplementary Figure 1 –ROI placement in epilepsy patients. 3](#_Toc194669464)

[Supplementary Figure 2 – Correlation between cSUV values and administered FDG activity per body weight. 3](#_Toc194669465)

[Supplementary Figure 3 – Effect of sex and puberty on cSUV values. 4](#_Toc194669466)

[Supplementary Figure 4 – Correlation between age at epilepsy onset and epilepsy duration and AI values calculated using SUV_max_ and SUV_mean_ values. 4](#_Toc194669467)

# Supplementary Table 1 – Different scanners used in the study

| **Scanner** | **Epilepsy**  **(n=29)** | **Non-epilepsy**  **(n=29)** |
| --- | --- | --- |
| Discovery RX | 5 | 12 |
| Discovery HR | 0 | 2 |
| Discovery LS | 0 | 1 |
| Discovery 690 | 2 | 1 |
| Discovery STE | 2 | 3 |
| Discovery MI | 9 | 2 |
| SIGNA | 11 | 8 |

# Supplementary Table 2 – Different reconstruction algorithms used in the study

| **Scanner** | **Epilepsy**  **(n=29)** | **Non-epilepsy**  **(n=29)** |
| --- | --- | --- |
| QCFX | 9 | 5 |
| Fore FBP 3D | 7 | 0 |
| VPFXS | 13 | 6 |
| 3D IR | 0 | 15 |
| OSEM | 0 | 3 |

# Supplementary Figure 1 –ROI placement in epilepsy patients.


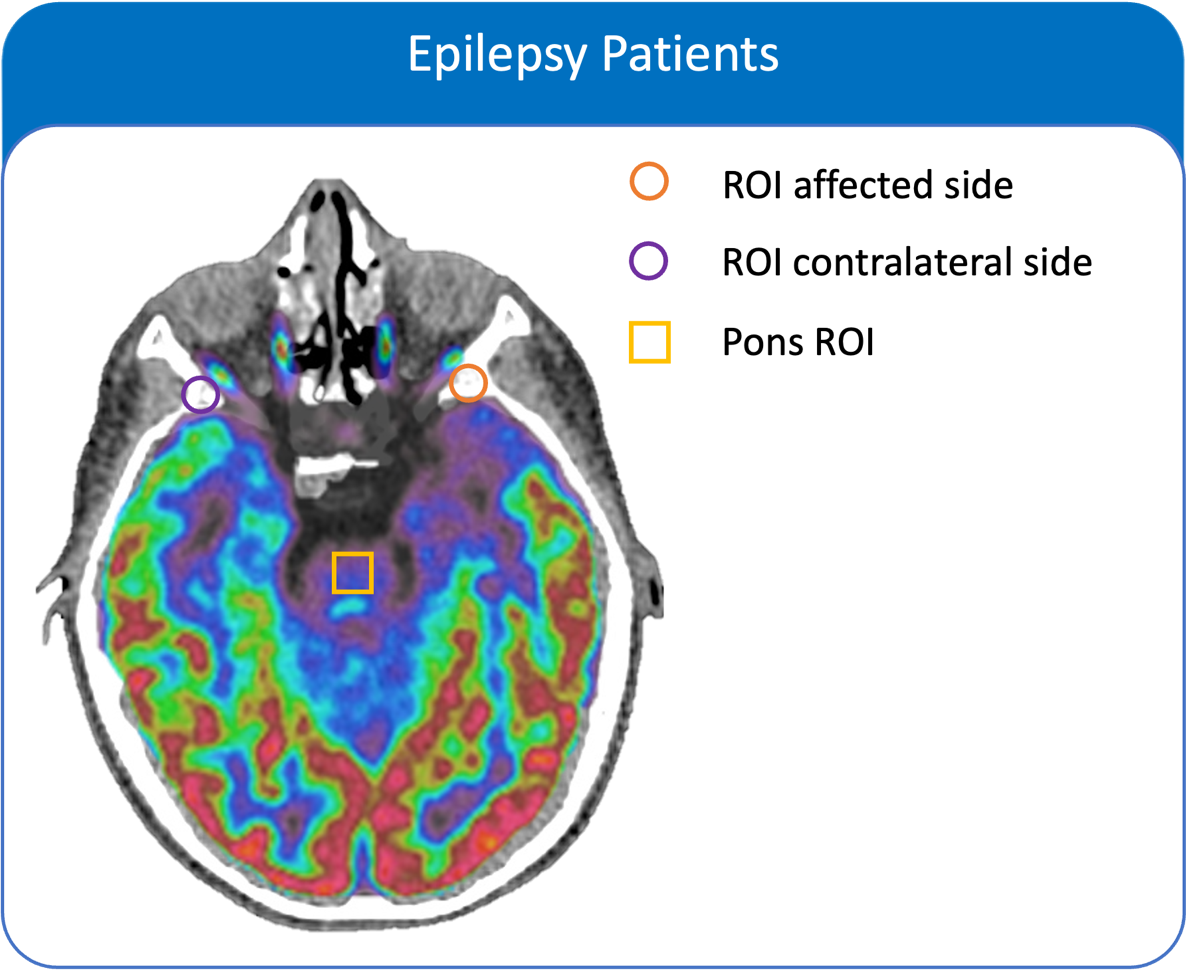


ROI: region of interest

# Supplementary Figure 2 – Correlation between cSUV values and administered FDG activity per body weight.


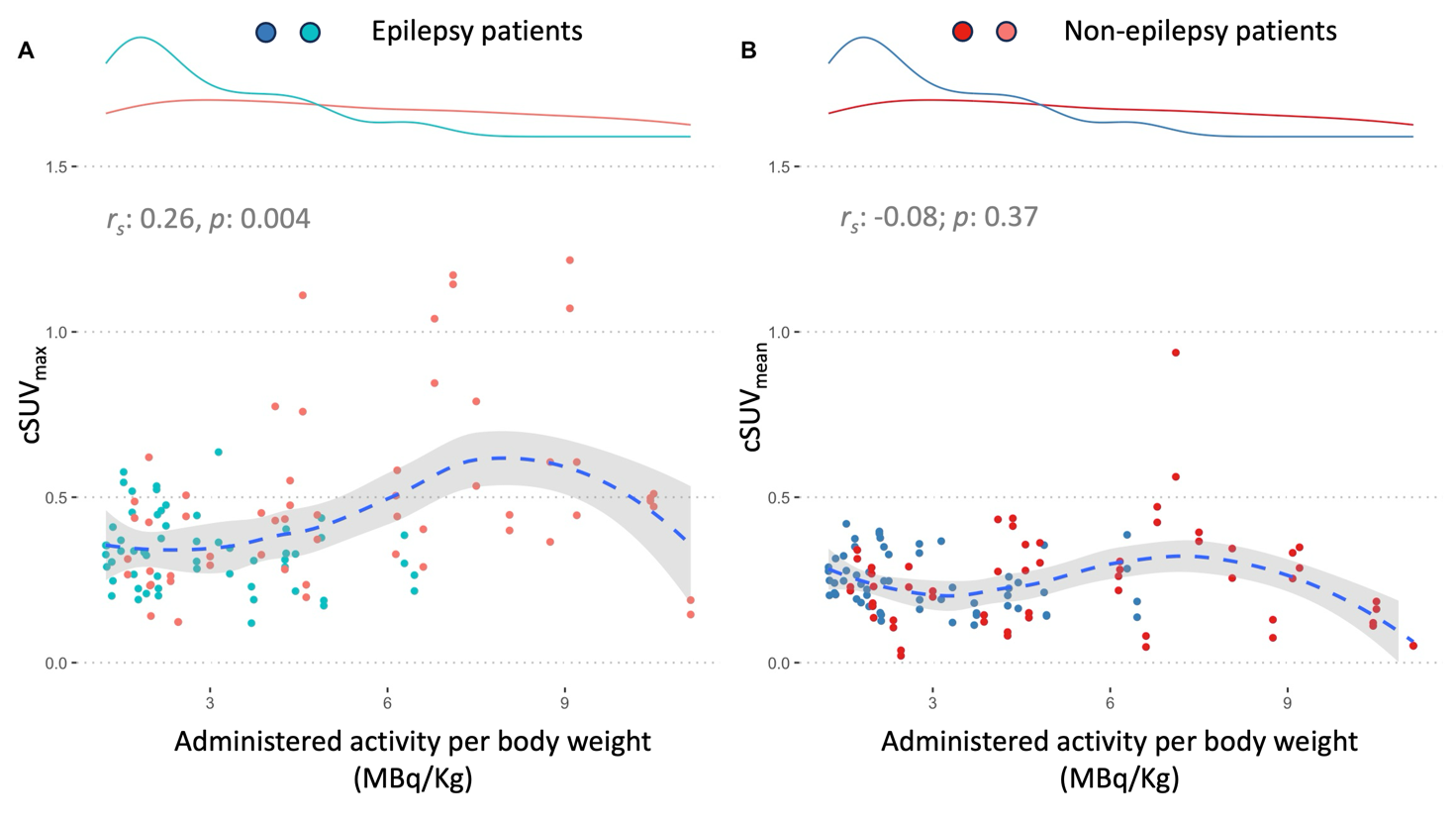


Scatter plots showing the correlation between cSUV and administered FDG activity per body weight. The density plots above indicate that FDG activity per body weight was higher in non-epilepsy than epilepsy patients. While cSUV values based on SUV_max_ correlated with FDG activity per body weight (A), those based on SUV_mean_ did not (B). The regression lines are modeled using a local polynomial regression, allowing local fitting, and evaluated with the Spearman correlation coefficient. Confidence intervals are represented in grey. FDG: 2-[^18^F]-fluoro-2-deoxy-D-glucose; cSUV: corrected standardized uptake value; MBq: mega becquerel

# Supplementary Figure 3 – Effect of sex and puberty on cSUV values.


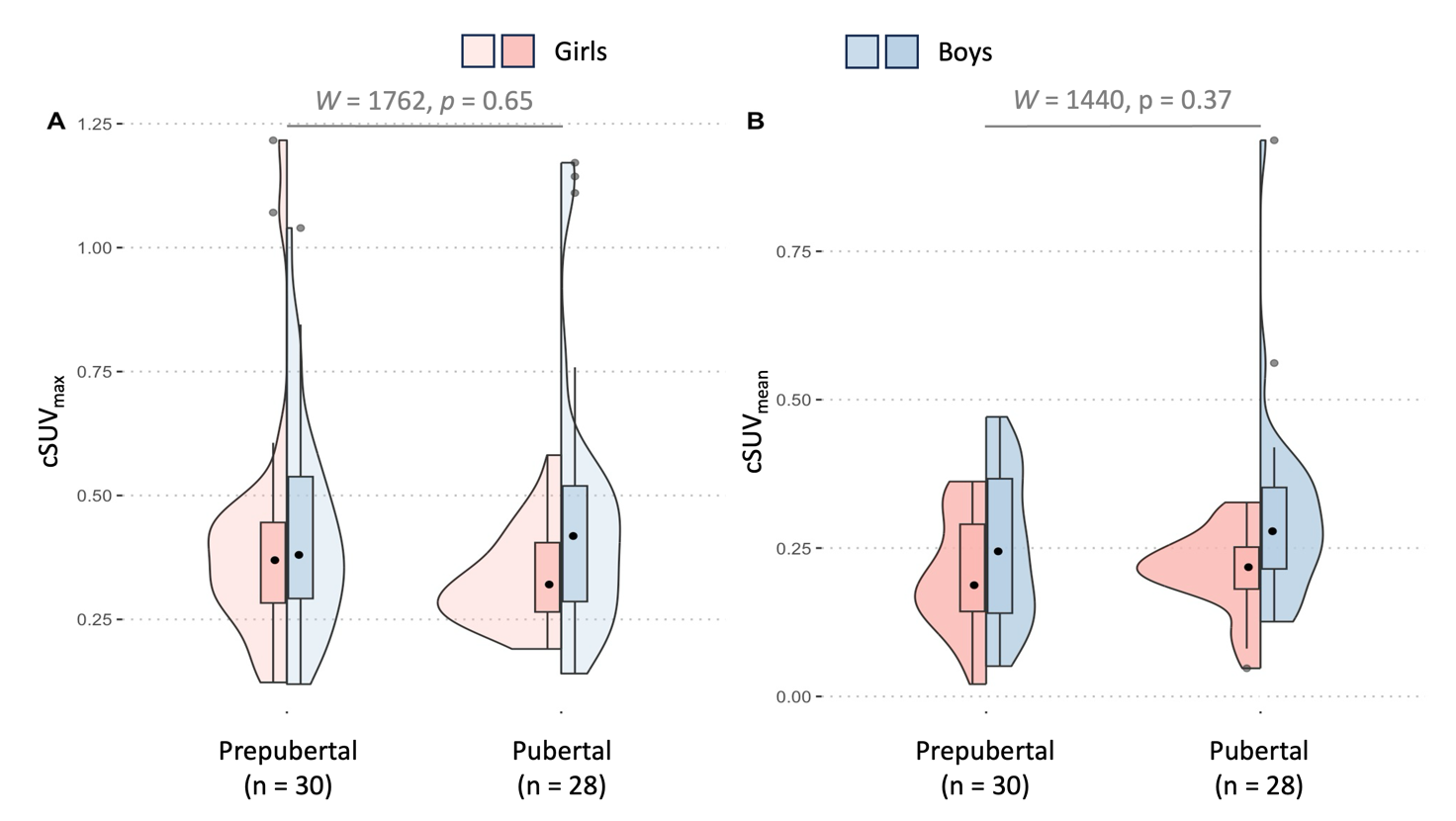


Violin and box plots detailing the overall distribution of (A) cSUVmax and (B) cSUVmean values between prepubertal (n = 30) and pubertal (n = 28) children (Wilcoxon-Mann-Whitney). Both prepubertal and pubertal cohorts were balanced between boys (n = 16 in both prepubertal and pubertal) and girls (n = 16 in prepubertal and n = 12 in pubertal). Each box plot presents a point representing the median cSUV value. cSUV: corrected standardized uptake value

# Supplementary Figure 4 – Correlation between age at epilepsy onset and epilepsy duration and AI values calculated using SUV_max_ and SUV_mean_ values.


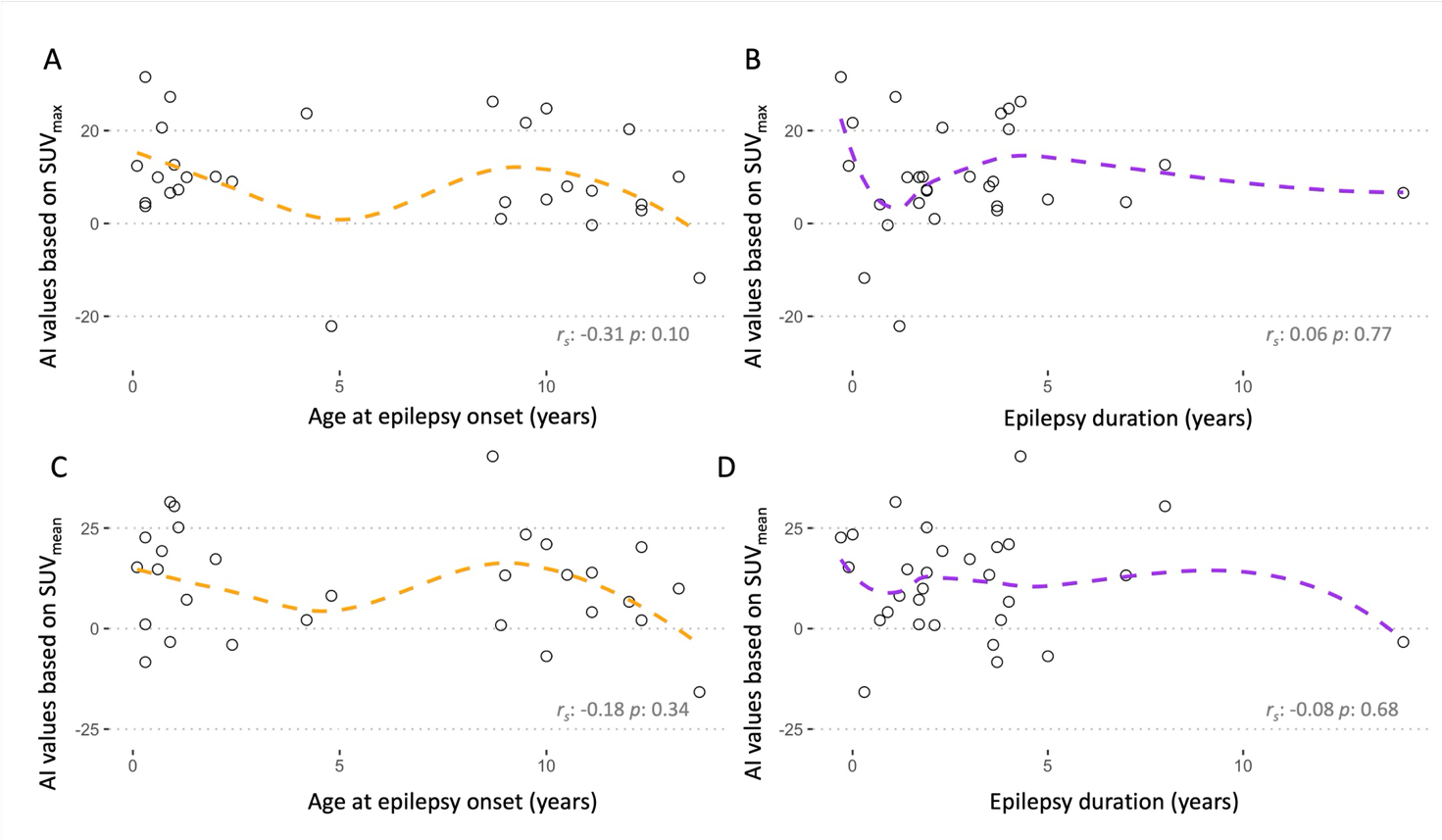
Scatter plots representing the absence of correlation between AI values, calculated using SUV_max_ (A, B) or SUV_mean_ (C, D), and age at epilepsy onset (A, C) or epilepsy duration (B, D), in epilepsy patients (represented as dots). The regression lines are modeled using a local polynomial regression, allowing local fitting. Considering the low numerosity of cases and the absence of a linear correlation between the variables, the Spearman correlation coefficient was deemed the correct statistical method to describe the data. AI: asymmetry index; SUV: standardized uptake value
